# Clinical application of multicolor optical coherence tomography in the diagnosis of retinal pathologies

**DOI:** 10.12669/pjms.346.16388

**Published:** 2018

**Authors:** Hunain Ghoghari, Syed Fawad Rizvi, Kaunain Razzak, Hina Loya

**Affiliations:** 1 *Dr. Hunain Ghoghari, MBBS. Layton Rahamatullah Benevolent Trust (LRBT) Free Base Eye Hospital, Korangi 2 ½, Karachi, Pakistan*; 2 *Prof. Syed Fawad Rizvi, MCPS (ophth) FCPS (ophth). Layton Rahamatullah Benevolent Trust (LRBT) Free Base Eye Hospital, Korangi 2 ½, Karachi, Pakistan*; 3 *Kaunain Razzak, Medical student (observer ship at LRBT) A-80, block 13-C, Gulshan-e-Iqbal, Karachi, Pakistan*; 4 *Dr. Hina Loya, MBBS Layton Rahamatullah Benevolent Trust (LRBT) Free Base Eye Hospital, Korangi 2 ½, Karachi, Pakistan*

**Keywords:** Multicolor OCT, Conventional color fundus photograph, vitreomacular interface disorder

## Abstract

**Objective:**

To assess the clinical application of multicolor optical coherence tomography (OCT) using confocal scanning laser ophthalmoscopy (cSLO) in different retinal pathologies.

**Methods:**

This observational study was conducted at the Layton Rahmatullah Benevolent Trust (LRBT), Free Base Eye Hospital, Karachi, from April 2018 to June 2018. It includes 36 patients suffering from different retinal pathologies including diabetic retinopathy, age related macular degeneration, and vitreomacular interface disorders using multicolor optical coherence tomography as a screening tool.

**Results:**

It was found that automated eye tracking system of this new version tool enables ophthalmologists to take high-resolution cSLO reflectance images. The light scatter can be avoided with the use of confocal optics. Appearances of pigment changes and hemorrhages were some of the differences found when compared to the conventional CFP. About 20% in AMD, 37.5% with diabetes and 100% patients with vitreomacular interface disorders could have be easily missed by CFP.

**Conclusions:**

Multicolor OCT can provide information and figures far more authoritatively than the conventional CFP, which is highly affected by media opacities. To interpret Multicolor OCT ophthalmologists should be watchful with plenty of understanding.

## INTRODUCTION

Imaging Techniques in the 21^st^ century have opened new avenues for ophthalmologists to diagnose and detect different diseases of the human eye, particularly the retina and manage them more efficiently. Digital Imaging as compared to the earlier film-based photography has transformed the modern ophthalmologic practices in Colour Fundus Photography (CFP), Fundus angiography (FA) and Optical Coherence Tomography (OCT).^1^ These advances have proved to be precise records for patients with various retinal pathologies which in the form of high-grade images can be instantly transferred to the concerned clinicians to be used for prompt decisions and guidance.^2^ The advantages that revolve around this modality are multiple with some being three-dimensional exploration, direct comparisons against former records and decrease patient follow up.^3^

The Color Fundus Photography (CFP) was one of the chief imaging modality throughout the 1800s, emerged as a successful tool to obtain pictures of the central and peripheral retina, macula and the optic disc in the 1950s. With the advent of modern technology, models with an intricate microscope attached to a flash enabled camera were constructed. Likewise improvements like digital imaging, stereoscopic imaging, non-mydriatic functions and wide-field technology have guaranteed its widespread clinical and screening use.^4^ Today Digital CFP is an essential section of any eye hospital’s diagnostics department, providing doctors with high quality duplicable images of retinal abnormalities which closely resemble with clinical examinations. However, computerized or semi-computerized analysis is made challenging and restricted with (1) the inconsistency of fundus pigmentation and illumination among different patients, (2) narrow-ranged resolution and contrast and (3) the utmost unfavorable media opacities.^5^

In 1980s optical instrument engineering progressed with the invention of Scanning Laser Ophthalmoscope (SLO). The alleged ‘flying spot TV ophthalmoscope’, enabled ophthalmologists with an alternate method of taking pictures of the fundus.^6^ SLO uses a series of vertical and horizontal scanning mirrors to inspect a particular region of the retina with a single point of laser light at a specified wavelength which display raster images on a television monitor. Confocal SLO (cSLO) systems provide a higher resolution and a better contrast by image procurement at various planes which suppresses light scatter. Additionally all the images can be obtained in real time via a non-mydriatic pupil which escalates patient compliance. The major drawbacks of SLO are (1) reflections from eye astigmatism and the cornea, (2) tainted data with eye movements during testing.^7^

Optical Coherence Tomography (OCT) is one the greatest imaging technique which uses coherent light to capture two and three dimensional images. The ‘Multicolor mode’ for the SPECTRALIS SD-OCT developed by Heidelberg engineering uses the basic cSLO technology mentioned above but with a few alterations. Three monochromatic laser sources replace the conventional single point of laser light and pick-up three simultaneous reflectance images. The core objective of using three wavelengths is to use their properties of penetrating and demonstrating the levels of the retina at different depths.

The Blue Reflectance (BR; 488nm) predominantly displays the particulars of the Inner Retina and the Vitreoretinal Interface such as the Macular pigment changes, Retinal nerve fiber layer thinning and the Epiretinal membranes.

The Green Reflectance (GR; 518nm) permit deeper penetration and the structures such as Retinal blood vessels and Intra retinal lipid exudation become visible.

The Infrared Reflectance (IR; 820 nm) visualizes the changes in Outer Retina and the Choroid including Drusen and Retinal Pigmentary Epithelium.^8^,^9^

## METHODS

This study was conducted in medical retina diagnostic department of Layton Rahmatullah Benevolent Trust (LRBT), Free Base Eye Hospital, Karachi, from April 2018 to June 2018 included 36 patients suffering from different retinal pathologies. In this study we used a different modality multicolor OCT for screening of different ocular pathologies such as Age related Macular Degeneration (AMD), Diabetic Retinopathy (DR), and viteromacular interface disorders and the effects they produce on the Retina and the Macula and show how changing wavelengths can present diverse retinal irregularities.

After obtaining approval from the Hospital Ethical Review Committee, a total of 36 patients (72 eyes) aged 40-60 years were included, 12 patients each with above mentioned disease. Informed consent was taken from all the included patients after explanation of the nature and possible consequences of the study.

Colour Fundus Photograph (CFP), was captured by means of a standard fundus camera (Nikon, D700, Japan) after dilatation of eyes with tropicamide 1.0% eye drops. The field of view was the usual at 30-40°, positioned on the macula. Spectralis OCT (Heidelberg Engineering) was used to perform high-speed combined and simultaneous cSLO + Spectral Domain-OCT (SD-OCT) imaging including Multicolor and the 70-kHz OCT prototype module device using the multicolor mode on an area of the central 30° and 55°. Image acquisition included Blue Reflectance – {BR (λ = 488 nm)}, near- Infrared Reflectance – {IR [λ = 820 nm]}, and Green Reflectance – {GR (λ = 518 nm)}.

## RESULTS

The Images were taken in patients who presented with AMD, DR and vitreomacular interface disorders. Patient’s mean age was 48.6 years in all three groups. Males were affected more commonly with DR, and vitreomacular disorders. While females (64%) were commonly affected with AMD. Retinal imaging of pathologies like AMD, DR, vitreomacular disorders can be better appreciated and visualized with the multicolor OCT.

### Age-Related Macular Degeneration

Today one of the main application of Multicolour OCT is in the context of Age-related Macular Degeneration (AMD) which has allowed ophthalmologists to identify it earlier and have it under regular observation during the period of anti-VEGF therapy. With Multicolor OCT imaging Reticular Pseudodrusen can be imaged and detected more distinctly when compared to the CFP.^10^ {Graham, 2017 #43} Also, the Geographic Atrophy (GA) borders are more clearly demarcated on the multicolor images than the CFP.^11^

During the research period 12 patients with decreased vision visited the medical retinal clinic of LRBT. There were eight females and 4 males. Out of the 12 patients three females had known bilateral disease therefore they were excluded from the study. The remaining nine patients went through standard screening procedure for eye signs, five of them showed clinical signs of AMD unilaterally and four had bilateral disease. Those who had unilateral involvement were investigated through both CFP, and multicolor OCT. One (20%) eye had enhanced visualization of early disease on OCT multicolor in comparison to CFP, which was easily missed on CFP.

**Fig.1:**
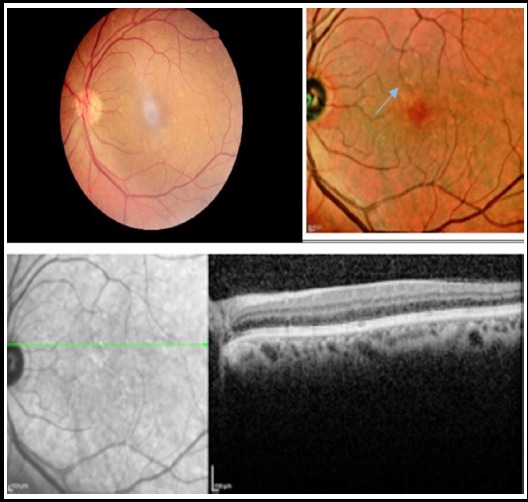
Early drusens (blue arrow) clearly visible on OCT multicolour and macular thickness.

**Fig.2:**
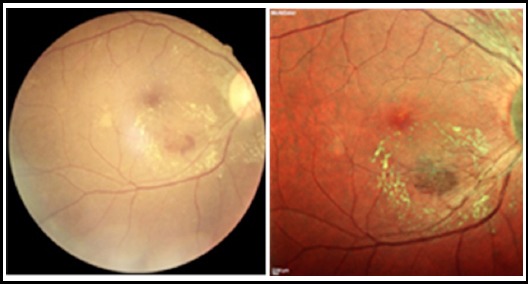
DR - retinal hemorrhage along with ERM appreciated more on multicolour OCT then CFP. (Hemorrhage shown by arrow).

### Diabetic Retinopathy

Patients and their families can greatly benefit from the early detection and screening of Diabetic Retinopathy. The multicolor images from the cSLO + SD-OCT platform facilitates ophthalmologists because it serves as a one-stop service for the patients, being a single device that can perform all the imaging modalities, so it is cost effective and also time saving.^12^,^13^ Twelve known diabetic patients for past four years, came for routine follow-up funduscopic examination. Seven were males, and five females. Out of which, one male and three females had bilateral diabetic retinopathy, which was easily picked clinically. Remaining 8 patients were found to have very mild non proliferative diabetic changes in one eye, whereas the other eye seem to be normal. These patients were investigated through CFP and multicolor OCT. Out of eight patients, three (37.5%) had early diabetic changes which were evident by multicolor OCT only. Hence, multicolor OCT can be the diagnostic tool for early screening of diabetic retinopathy.

**Fig.3:**
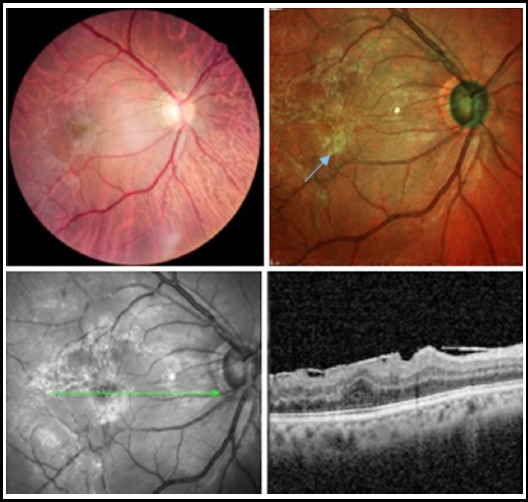
excellent imagining of ERM (blue arrow) on multicolour imaging.

### Vitreomacular interface Disorders

With the advent of the multicolour OCT the detection of vitreomacular traction have become very easy for ophthalmologists as compared to the conventional CFP.

Twelve patients came with blurring and distortion of vision. Nine were males, and three females. Out of which three females had diabetic vitreomacular traction, and two male patients had incomplete posterior vitreous detachment. But remaining seven male patients had no obvious clinical signs of any pathology. These seven patients went through CFP and OCT multicolor, and were found to have grade 0 epiretinal membrane (ERM). The grading and extent of ERM was better visualized by Multicolor OCT in all the seven patients (100%).

## DISCUSSION

Retinal diseases can be screened, documented and managed with the help of high-grade fundus imaging. The Multicolour mode on the combined cSLO + SD-OCT platform is a big step forward in ophthalmic practices enabling these fundus images to be taken with a solitary device which eliminates the need of colour fundus cameras or any supplementary appliances. Moreover, this setup has the advantage of being used to capture multimodal images such as fundus autofluorescence, infra-red imaging, SD-OCT, fluorescein and indocyanine green angiography.^1^,^2^,^14^ Also, the peripheral changes can be viewed by capturing 55° images using different lens attachments. In smaller ophthalmic set-ups where cost and space restrictions allow limited equipment, this device proves very useful screening tool furthermore it saves a lot of time in hospitals and clinics with a high patient turnover.^15^

One of its core features is that small laser spots with single-wavelengths substitute the bright flash of light necessary in the CFP which causes far less photophobia and escalates patient obedience. The multicolour image is made up of three reflectance images which can be viewed separately to enable the ophthalmologist to study the details of different layers of the retina and detect pathologies associated with the structures irrespective of each other.^16^

Muftuoglu et al, in his study of visualization of macular pucker by multicolour imaging, showed that 56.2% ERM was better visualized by using multicolour imaging than CFP.^17^ Similar results, in favor of multicolour OCT in detection of ERM was also demonstrated by L Reznicek et al.^18^ Similarly, a study was conducted by Ben Moussa N et al, which stated that multicolour OCT have proved to be an excellent tool for measurement of geographic atrophy.^19^ Furthermore, a study Graham KW et al in 2017 also presented results in favor of multicolour imaging in detection of pigment clumping (69.7%) to >80% detection of atrophic patches of AMD.^10^

Ahmed MSZ et al, in their study of multicolour imaging for detection of diabetic retinopathy, also stated the benefits of using this newer modality.^12^ Li S et al, stated that multicolour imaging is superior to CFP, in resolution and better visualization of micro aneurysms, diabetic macular edema, and ERM, though cotton wool spots, hemorrhages, exudates were not significantly appreciated with multicolour imaging.^13^

In summary, conventional CFP can be replaced by multicolour imaging as it has many applications. With the widespread use of multicolour imaging in recent times, further studies have become compulsory to authenticate the retinal details on the multicolour imaging with the conventional CFP.
